# Establishment of a Fah-LSL mouse model to study BEC-to-hepatocyte conversion

**DOI:** 10.52601/bpr.2023.230034

**Published:** 2023-12-31

**Authors:** Xingrui Wang, Wenjuan Pu, Huan Zhu, Mingjun Zhang, Bin Zhou

**Affiliations:** 1 New Cornerstone Science Laboratory, State Key Laboratory of Cell Biology, Shanghai Institute of Biochemistry and Cell Biology, Center for Excellence in Molecular Cell Science, Chinese Academy of Sciences, University of Chinese Academy of Sciences, Shanghai 200031, China; 2 Key Laboratory of Systems Health Science of Zhejiang Province, School of Life Science, Hangzhou Institute for Advanced Study, University of Chinese Academy of Sciences, Hangzhou 310024, China; 3 School of Life Science and Technology, ShanghaiTech University, Shanghai 200031, China

**Keywords:** Liver regeneration, BEC-to-hepatocyte transdifferentiation, Fumarylacetoacetate (*Fah*) gene, Transitional liver progenitor cells (TLPC), Lineage tracing

## Abstract

The liver consists predominantly of hepatocytes and biliary epithelial cells (BECs), which serve distinct physiological functions. Although hepatocytes primarily replenish their own population during homeostasis and injury repair, recent findings have suggested that BECs can transdifferentiate into hepatocytes when hepatocyte-mediated liver regeneration is impaired. However, the cellular and molecular mechanisms governing this BEC-to-hepatocyte conversion remain poorly understood largely because of the inefficiency of existing methods for inducing lineage conversion. Therefore, this study introduces a novel mouse model engineered by the Zhou's lab, where hepatocyte senescence is induced by the deletion of the fumarylacetoacetate (*Fah*) gene. This model facilitates the efficient conversion of BECs to hepatocytes and allows for the simultaneous lineage tracing of BECs; consequently, a transitional liver progenitor cell population can be identified during lineage conversion. This study also outlines the technical procedures for utilizing this model to determine the underlying cellular and molecular mechanisms of BEC-to-hepatocyte conversion and provides new insights into liver regeneration and its underlying molecular mechanism.

## INTRODUCTION

The liver is the largest internal organ in the body and performs many vital functions. Most of the liver mass consists of two epithelial cell populations, namely, hepatocytes and biliary epithelial cells (BECs; also termed cholangiocytes). Hepatocytes process and absorb nutrients, produce serum proteins, and metabolize pharmaceutical drugs and toxins. BECs, which form bile duct tubules, are responsible for the modification and transportation of bile along the biliary tree into the small intestine (Gadd *et al.* 2020). Therefore, a functional hepatocyte pool should be maintained during homeostasis or in response to injury (Michalopoulos 2007, 2021; Michalopoulos and DeFrances 1997; Miyajima *et al.* 2014).

Previous genetic lineage tracing studies demonstrated that the hepatocyte pool is mainly replenished through the self-renewal of pre-existing hepatocytes rather than differentiation from liver stem/progenitor cells during homeostasis and injury conditions (Schaub *et al.* 2014; Yanger *et al.* 2014). However, BECs can function as facultative liver progenitor cells (LPCs) and transdifferentiate into functional replication-competent hepatocytes when hepatocytes become senescent and hepatocyte-mediated liver regeneration is impaired in mice (Español-Suñer *et al.* 2012; Shin *et al.* 2015). In terms of inducing liver injury, Forbes lab knocked down β1-integrin or overexpressed p21 to inhibit hepatocyte proliferation (Raven *et al.* 2017); Xie’s lab used long-term thioacetamide treatment to induce chronic liver injuries (Deng *et al.* 2018). They observed that BECs can transdifferentiate into hepatocytes and contribute to liver regeneration. Nevertheless, the cellular mechanism through which BECs differentiate into hepatocytes via a hepatic progenitor cell state and the molecular mechanisms by which BECs differentiate into hepatocytes remains unclear. One significant reason for this knowledge gap is the low efficiency of previously reported methods for inducing the conversion of BECs to hepatocytes; consequently, it has hindered in-depth studies on cellular and molecular mechanisms.

Recently, Zhou’s lab generated a mouse model, where the fumarylacetoacetase (*Fah*) gene is deleted, thereby causing hepatocyte senescence (Pu *et al.* 2023). This model can stably and efficiently induce BEC-to-hepatocyte conversion. Combining this model with lineage tracing techniques, we could identify a subset of bipotent transitional liver progenitor cells (TLPCs) that originated from BECs and determine the cellular and molecular mechanisms. Here, we provided the technical details for constructing BEC-to-hepatocyte conversion mouse models and used this model to further characterize TLPCs.

### Development of the protocol

To study BEC-to-hepatocyte transdifferentiation during liver injury, we generated a mouse model, *Fah-LSL*, where *Fah* is deleted; thus, hepatocyte senescence occurred during liver regeneration, human hereditary tyrosinemia type I caused by FAH deficiency was modeled (Nobili *et al.* 2010), and BEC-to-hepatocyte transdifferentiation was induced. By crossing *Fah-LSL* with an *ACTB-Cre* mouse, we examined whether *Fah* was not expressed in *Fah-LSL/LSL* mice and whether *Fah-LSL/LSL*;*ACTB-Cre* mice restored their *Fah* expression. Next, we generated *CK19-CreER*;*Fah-LSL/LSL*;*R26-tdT* mice, whose tdTomato (tdT) was induced and *Fah* was restored in BECs after tamoxifen (TAM)-induced Cre-loxP recombination. *CK19-CreER* specifically targets BECs but not hepatocytes (Means *et al.* 2008). Lineage tracing reveals that the loss of FAH function and associated liver injury is necessary to induce BEC-to-hepatocyte transdifferentiation. Lastly, we generated *CK19-CreER*;*HNF4α-DreER;Fah-LSL/LSL;R26-RL-tdT* mice, whose tdT could be only expressed after the TAM-induced removal of two Stop sequences by both Dre-rox (HNF4α+) (Han *et al.* 2019; Thakur *et al.* 2019) and Cre-loxP (CK19+) recombinations; we also characterized BEC-derived transitional liver progenitor cells (TLPCs) by immunostaining and clonal analysis.

### Applications and advantages of the protocol

The discovery of BEC-to-hepatocyte transdifferentiation during the impairment of hepatocyte-mediated regeneration provided an important new concept enabling liver regeneration. Unfortunately, the cellular identity of BECs with such facultative LPC potential and the molecular mechanisms enabling their transdifferentiation remained unclear. This protocol provided a complete workflow of constructing mouse models to study BEC-to-hepatocyte conversion in severe liver injury. Our model showed remarkable advantages in inducing TLPCs compared with other published models; using this model, researchers could trace TLPCs stably and efficiently. Thus, it would lay a foundation for subsequent single-cell transcriptomic analysis or other analytical procedures.

### Limitations of the protocol

Mouse generation is the first and most fundamental step in this protocol. In the absence of relevant experience or proper techniques, it is time consuming. It also extends the necessary working time if the wanted allele of gene is not inherited by descendants during mouse breeding.

### Overview of the protocol

**Figure 1:**
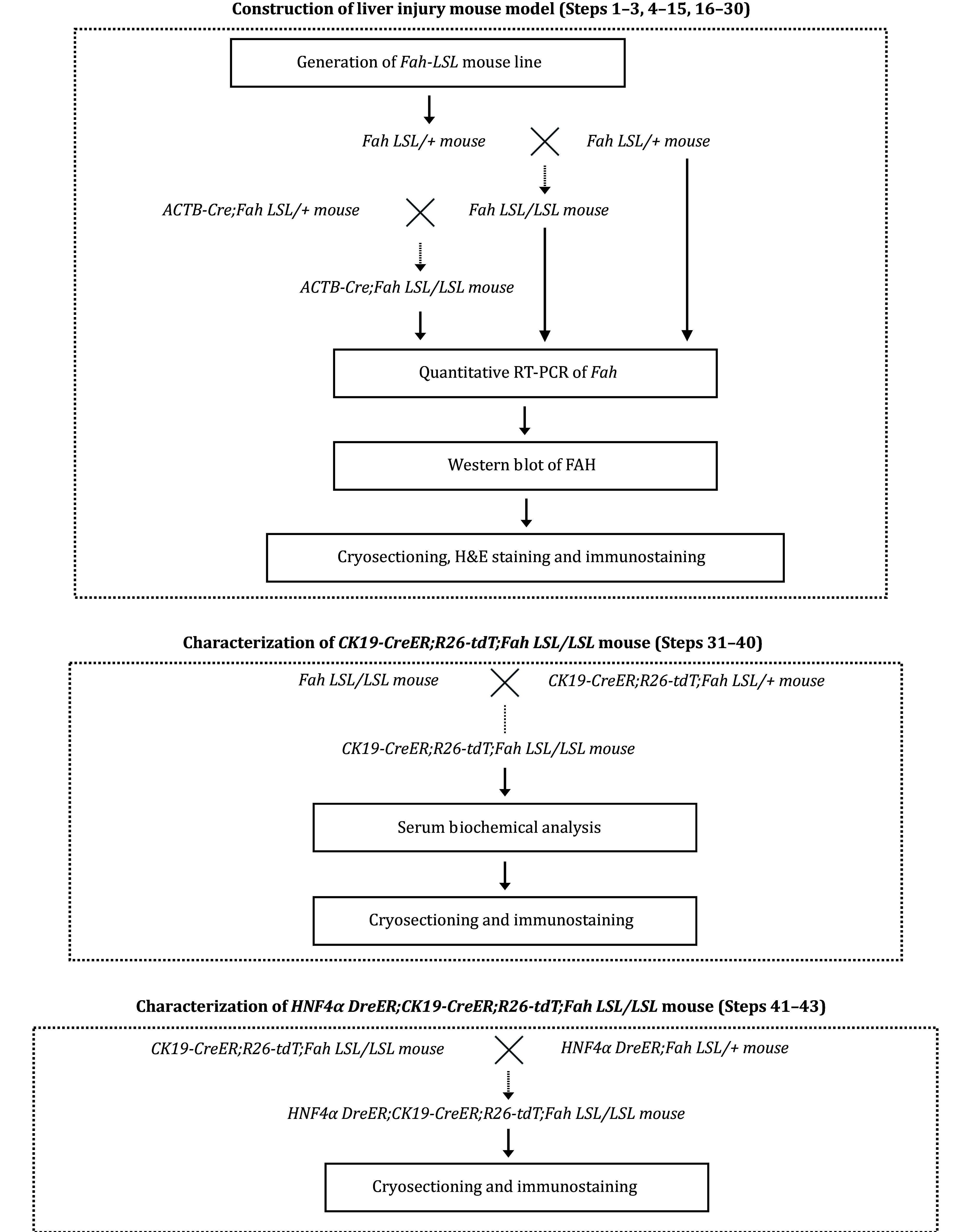
Strategy of crossing mice for the purpose of experiment. First, *Fah-LSL* homozygous mice was constructed. *Fah-LSL/LSL* mice and *Fah-LSL/LSL;ACTB-Cre* mice were generated through mating. By examing the Fah expression and phenotypes of the mice, the liver injury mouce model was validated. Next, to trace BECs in the liver injury model, *CK19-CreER;Fah-LSL/LSL;R26-tdT* mice were generated, and their tdT was induced and Fah was restored in BECs after tamoxifen-induced Cre-loxp recombination. Lastly, a Cre-loxp and Dre-rox based dual genetic lineage tracing approach to idelibly label CK19+ HNF4α+ TLPCs was developed to further characterize BEC-derived TLPCs.

The construction of a feasible mouse model for studying BEC-to-hepatocyte conversion during severe liver injury involves three parts (see strategies for crossing mice and corresponding experiments in Fig. 1). First, we generated the *Fah-LSL* mouse line showing hepatocyte senescence, which can be induced. By crossing it with an *ACTB-Cre* mouse, we validated that our genetic strategy worked. Next, we bred *CK19-CreER*;*Fah-LSL/LSL*;*R26-tdT* mice to trace the behavior of BECs during liver injury. Lastly, dual genetic lineage tracing was implemented to comprehensively understand TLPC with multiple phenotypic analysis.

## SUMMARIZED PROCEDURE

### Generation of mouse lines (*Fah-LSL* mouse line)

1　Insert the loxp-stop-loxp sequence into the 3ʹ-untranslated region of *Fah* to generate the *Fah*-*LSL* mouse line by embryonic stem cell targeting.

2　Screen and breed chimeric mice for further verification.

### Characterization of *Fah-LSL* mouse line

1　Cross *Fah*-*LSL*/+ mice with *Fah*-*LSL*/+ mice to obtain *Fah-LSL/LSL* mice. Cross *Fah*-*LSL*/+ mice with *Fah*-*LSL*/+; *ACTB-Cre* mice to obtain *Fah-LSL*/*LSL*;*ACTB-Cre* mice.

2　Treat all *Fah-LSL*/*LSL* mice with 7.5 μg/mL 2-(2-nitro-4-trifluoro-methylbenzoyl)-1,3-cyclohexanedione (NTBC)-containing water. NTBC is a drug that can prevent injury in hepatocytes with *FAH* deletion.

3　Analyze the relative mRNA expression levels of *Fah* in the livers of adult *Fah-LSL*/+, *Fah-LSL*/*LSL* and *Fah-LSL*/*LSL*;*ACTB-Cre* mice.

4　Perform quantitative RT-PCR on QuantStudio 6 Real-Time PCR System. Generate a melt curve by using the Applied Biosystems real-time PCR system software. Compare the CT of an unknown sample against a standard curve with known copy numbers to obtain absolute quantitation.

5　Analyze the relative protein expression levels of Fah in *Fah-LSL*/+, *Fah-LSL*/*LSL*, and *Fah-LSL*/*LSL*;*ACTB-Cre* mice by using Western blot.

6　Maintain *Fah-LSL*/*LSL* mice on NTBC-supplemented water until they are eight weeks old. Terminate NTBC administration and measure the body weight of *Fah-LSL*/*LSL* mice and *Fah-LSL*/+ littermates after NTBC withdrawal. Normalize data to the body weight at week 0 (*n* = 5 mice).

7　Conduct a histological analysis of the livers of *Fah-LSL*/+, *Fah-LSL*/*LSL,* and *Fah-LSL*/*LSL*;*ACTB-Cre* adult mice to assess hepatic architecture and identify any extensive liver damage throughout the liver lobules.

### Characterization of *CK19-CreER;R26-tdT;Fah-LSL* mouse line

1　Cross the *CK19-CreER*;*R26-tdT* mouse line with the *Fah-LSL*/*LSL* line to trace BECs during liver injury.

2　In this assay, supply NTBC-containing water (7.5 μg/mL) for eight weeks after birth. Administer 0.2 mg TAM per gram mouse body weight via oral gavage four times 14 days before NTBC withdrawal, and administer an equal amount of oil to the control group. Then, 28 days after NTBC withdrawal, reintroduce NTBC for five days.

3　Conduct serum biochemical analysis for liver function. Collect blood from the indicated mice and centrifuge at 850 *g* and 4 °C for 15 min. Compare ALT and AST levels under different conditions (TAM or oil administration).

4　Collect samples at the designed experimental time for cryosection and staining. Collect the samples on the positively charged slides. Perform immunofluorescence staining based on the antibodies organized in the materials.

### Dual genetic lineage tracing for the analysis of CK19+ HNF4α+ TLPCs

1　Cross the *CK19-CreER*;*Fah-LSL*/*LSL;R26-RL-tdT* mouse line with the *HNF4a-DreER;Fah-LSL*/*LSL;* mouse line to further analyze the characterization of TLPCs.

2　In this assay, administer a low dosage of TAM (0.05 mg TAM per gram mouse body weight) 20 days after NTBC removal. Collect samples 25 days after NTBC removal, and confirm whether R26-RL-tdT could only be activated by dual recombination.

3　Collect samples at the designed experimental time for cryosection and immunostaining.

4　Perform clonal analysis. After low-dosage TAM administration, conduct sparse labelling and clonal analysis at seven weeks post low-dose TAM.

## EXPERIMENTAL DESIGN

### Mouse generation

Experiments involving mice were executed in strict compliance with the protocols sanctioned by the Institutional Animal Care and Use Committee (IACUC) at the Center for Excellence in Molecular Cell Science, a division of the Shanghai Institutes of Biological Sciences under the Chinese Academy of Science. The ethical approval for these animal studies was granted under the protocol number SIBCB-S374-1702-001-C1. In this procedure, the *Fah-LSL* mouse line employed was engineered through homologous recombination. The *CK19-CreER*, *HNF4α-DreER*, *R26-tdT*omato (*R26-tdT*), and *R26-RL-tdTomato* (*R26-RL-tdT*) mouse lines were reported previously (Han *et al.* 2019; Madisen *et al.* 2010; Means *et al.* 2008). Male and female mice, aged 8–20 weeks, were used in the experiments with similar-aged mice for both control and experimental groups. All mice were housed at the Laboratory Animal Center of the Center for Excellence in Molecular Cell Science in a specific pathogen-free (SPF) facility with individually ventilated cages. The room had controlled temperature (20–25 °C), humidity (30%–70%), and light (12 h light–dark cycle).

### Mouse genotyping

When a single-genotype mouse line was generated, mice carrying multiple genotypes were obtained by breeding with other mice. The genotypes of the newborn offspring were identified for later experiments. The toes of the mice were cut 7–10 days after birth while the mice were being marked. Through lysis, DNA precipitation, and DNA dissolution, DNA templates were prepared for PCR. With specific paired primers, results could be distinguished from specific bands after electrophoresis.

### RNA isolation and quantitative RT-PCR

Total RNA was extracted from the liver of the indicated mice or BECs were isolated from the indicated mice treated with TAM or oil. Cells were lysed with Trizol, and RNA was extracted in accordance with the manufacturer’s protocol. After RNA isolation, it was reverse-transcribed to generate complementary DNA (cDNA) by using the Prime Script RT kit. Quantitative reverse-transcription polymerase chain reaction (qRT-PCR) was executed with the SYBR Green qPCR master mix on a QuantStudio 6 Real-Time PCR System. GADPH was used as the endogenous control for normalization. For the quantification of *Fah* gene expression, forward and reverse primers were designed to anneal to exon 7 and exon 8, respectively; as a result, a 74 base-pair amplicon spanning these exons was obtained.

### Western blot

Liver tissues were harvested and homogenized in RIPA lysis buffer with protease inhibitors. After centrifugation, the supernatant was collected, and the protein concentration was quantified. The samples were denatured and subjected to gel electrophoresis using precast gradient gels. Proteins were transferred to polyvinylidene fluoride membranes, which were then blocked and incubated with primary antibodies for FAH and GAPDH. After the membranes were washed and incubated with HRP-conjugated secondary antibodies, chemiluminescent signals were captured using an imaging system.

### Serum biochemical analysis

Blood samples were obtained from the selected mice and centrifuged at 850 *g* and 4 °C for 15 min to isolate serum, which was then biochemically analyzed. Alanine aminotransferase (ALT) and aspartate aminotransferase (AST) levels were assessed using a VITROS 4600 fully automatic biochemical analyzer.

### Immunostaining

Liver specimens were fixed in 4% paraformaldehyde at 4 °C for 1 h, subsequently washed in phosphate-buffered saline (PBS), and dehydrated in a 30% sucrose solution at 4 °C overnight. The samples were then embedded in an optimal cutting temperature (OCT) compound. Cryosections were washed with PBS and incubated in a blocking buffer containing 5% normal donkey serum and 0.1% Triton X-100 at ambient temperature for 30 min. Sections were stained with primary antibodies and incubated overnight at 4 °C. Fluorescent signals were elicited using Alexa-conjugated secondary antibodies. For signal amplification, horseradish peroxidase (HRP)-conjugated antibodies in conjunction with a tyramide signal amplification kit were used.

### H&E staining

Cryosections were initially washed in PBS for 5 min to eliminate the residual OCT compound. Subsequently, sections were stained with hematoxylin A for 10 min and rinsed with distilled water. The samples were then treated with a 1% hydrochloric acid solution, diluted in 70% ethanol for 1 min, and thoroughly rinsed with water. Neutralization was achieved by incubation in 1% ammonia water for 1 min; then, the samples were rinsed with water. Sections were then exposed to Eosin-Y staining for a brief 10 s interval. A dehydration process involving a series of ethanol and xylene washes was applied. Ultimately, the sections were mounted using a resinous medium, and microscopic analysis was conducted using an Olympus BX53 microscope.

## PROCEDURE

### Generation of mouse lines (*Fah-LSL* mouse line) [TIMING ~6 months]

1　Choose a proper knock-in site on the targeted gene. The information about *Fah* can be found at http://www.ensembl.org/Mus_musculus/Gene/Summary?g=ENSMUSG00000030630;r=7:84585159-84606722

2　Construct the targeting vector containing the loxp-stop-loxp sequence and other indispensable elements.

3　Through targeting embryonic stem cells, insert the loxp-stop-loxp sequence between exon1 and exon2 of *Fah* via homologous recombination. After chimeric mouse screening, obtain the *Fah-LSL* mouse line (Shanghai Model Organisms Center, Inc.)

### Genotyping of knock-in mouse lines [TIMING ~17 h]

4　Design the genotyping primers (the primers used for different alleles are provided in Table 1) by using the Primer-BLAST function on the National Center for Biotechnology Information website (https://www.ncbi.nlm.nih.gov/tools/primer-blast/).

**Table 1:** Genotyping and qRT-PCR primers

Gene name	Primer sequences	Product size
Mouse line		
*CK19-CreER*	Forward: GCAGAATCGCCAGGAATTGACC	Mut: 300 bp; WT: no band
	Reverse: GTTCTTGCGAACCTCATCACTC	
*Fah-LSL*	Forward: GCTTTCTTGTCCTCTGTTCTCGTTC	Mut: 828 bp; WT: no band
	Reverse: TTTCAGTTAGCCTCCCCCCTACTC	
*R26-tdT*	Forward: GGCATTAAAGCAGCGTATCC	Mut: 196 bp; WT: no band
	Reverse: CTGTTCCTGTACGGCATGG	
*HNF4a-DreER*	Forward: GCTCTGGTGGTCTGCTCTGA	Mut: 626 bp; WT: no band
	Reverse: CCCTTGTTGAATACGCTTGA	
*R26-RL-tdT*	Forward: ACGGGTGTTGGGTCGTTTGTTC	Mut: 404 bp; WT: no band
	Reverse: ATGTTTCAGGTTCAGGGGGAGGTG	
qRT-PCR		
*Fah*	Forward: CCTGCAGACTCTTAGACATGGA	
	Reverse: GATTGGCTCTCCGAATCTGT	
*GAPDH*	Forward: TTGTCTCCTGCGACTTCAAC	
	Reverse:GTCATACCAGGAAATGAGCTTG	

5　Cut ~0.1 cm toes from the mouse 7–10 days after birth.

6　Add 250 μL of lysis buffer with proteinase K (100 μg/mL) to each 1.5 mL centrifuge tube containing the toes. Incubate the mixture at 60 °C overnight.

7　Vortex the tube to suspend the precipitate thoroughly and then centrifuge the tube at maximum speed (~15,000 *g*) at 25 °C for 7 min.

8　Mix the supernatant with 250 μL of isopropanol in a new 1.5 mL centrifuge tube.

9　Centrifuge the sample at maximum speed (~15,000 *g*) at room temperature for 3 min.

10 Discard the supernatant and then add 700 μL of 70% ethanol.

11 Centrifuge the sample at maximum speed (~15,000 *g*) at room temperature for 3 min.

12 Discard the supernatant and air-dry the DNA precipitate at 60 °C for 1–2 h.

13 Dissolve the DNA in 100–200 μL of distilled water.

14 Set up the PCR reaction mix as detailed in Table 2. Run the PCR in an optimized program. The recommended annealing temperature is 60 °C with 30 PCR cycles.

**Table 2:** Reaction composition

Genotyping PCR			Reverse-transcription reaction	
Component	Volume (µL)		Reagent	Volume (µL)
2× Taq PCR mix	5		Reaction solution from Step 24	10
Genomic DNA (100–200 ng/uL)	1		5X PrimeScript Buffer 2 (for real time)	4
Primer pairs (forward and reverse, 2.5 mmol/L)	0.8		PrimeScript RT Enzyme Mix I	1
Distilled water	3.2		RT Primer Mix	1
Total	10		RNase free dH_2_O	4
			Total	20

15 Run the PCR products in a 1% agarose gel at 150 V for 30 min and then use the UV detector to capture the image and analyze the results.

**[?TROUBLESHOOTING]**

### Characterization of the *Fah-LSL* mouse line

16 Mate the mice when they are sexually mature (6–8 weeks old) and strong enough. According to our IACUC, two female mice and one male mouse in one mating cage are allowed to prevent crowded cages. Cross *Fah*-*LSL*/+ mice with *Fah*-*LSL*/+ mice to obtain *Fah-LSL/LSL* mice. Cross *Fah*-*LSL*/+ mice with *Fah*-*LSL*/+;*ACTB-Cre* mice to obtain *Fah-LSL*/*LSL*;*ACTB-Cre* mice.

17 Treat all *Fah-LSL/LSL* mice with 7.5 μg/mL NTBC-containing water.

**[TIP]** Without NTBC, *Fah-LSL/LSL* mice cannot reach adulthood (Grompe *et al.* 1993).

### RNA extraction [TIMING ~3 h]

18 Analyze the relative mRNA expression levels of *Fah* in the livers of adult *Fah-LSL/+*, *Fah-LSL/LSL*, and *Fah-LSL/LSL*;*ACTB-Cre* mice. Cut a liver tissue into proper size. Add 1 mL of TRIzol reagent (Invitrogen, 15596018) per 50–100 mg of tissue. Incubate for 5 min to allow the complete dissociation of the nucleoprotein complex.

19 Add 0.2 mL of chloroform per 1 mL of TRIzol reagent used for lysis, securely cap the tube, and thoroughly mix by shaking. Incubate for 2–3 min. Centrifuge the sample at 12,000 *g* at 4 °C for 15 min.

20 Transfer the RNA-containg aqueous phase to a new tube. Add 0.5 mL of isopropanol to the aqueous phase per 1 mL of TRIzol reagent used for lysis. Incubate at 4 °C for 10 min.

21 Centrifuge at 12,000 *g* and 4 °C for 10 min to precipitate the RNA. Discard the supernatant with a micropipette.

22 Wash the RNA with 75% ethanol at 1 mL per milliliter of TRIzol reagent used for lysis. Air-dry the RNA pellet for 5–10 min.

**[?TROUBLESHOOTING]**

23 Resuspend the pellet in 20–50 µL of RNase-free water, 0.1 mmol/L EDTA, or 0.5% SDS solution by pipetting up and down. Incubate in a water bath or heat the block set at 55–60 °C for 10–15 min. Proceed to downstream applications, or store the RNA at –70 °C.

24 Prepare a master mix for genomic DNA elimination: 5× gDNA Eraser Buffer 2.0 μL; gDNA Eraser 1.0 μL; and total RNA 1 μg. Add RNase-Free dH_2_O to a total of 10.0 μL. Incubate at room temperature for 30 min.

### Reverse-transcription reaction and quantitative PCR [TIMING ~3 h]

25 Prepare a reverse-transcription reaction solution on ice according to Table 2. Incubate initially at 37 °C for 15 min and then at 85 °C for 5 s; store at 4 °C. The reagents used in Steps 24 and 25 are from the Prime Script RT kit (Takara, RR047A).

26 Perform Quantitative RT-PCR on QuantStudio 6 Real-Time PCR System (Thermo Fisher Scientific). Hold at 95 °C for 10 min, then go to 40 cycles, including denaturation at 95 °C for 15 s and annealing at 60 °C for 1 min. Use two-step RT-PCR with Power SYBR Green PCR Master Mix (2X; Thermo Fisher Scientific, 4367659). Check the plate configuration for optimization on the manuscripts. For qPCR of *Fah*, the forward primer for qPCR is located in exon7 and the reverse primer is located in exon8, and the PCR produced is 74 bp overlapping part of exon7 and exon8. The sequences of all primers are included in Table 3.

**Table 3:** Troubleshooting list

Step	Problem observed	Possible reason	Solutions
15	There may be unexpected non-specific bands	The paired primers are not designed to be specific to the whole genome.	When designing primers using NCBI website, make sure to use the recently-updated mouse genome as an reference
22	Yield RNA is hard to dissolve	RNA pellet is over-dry	Control the time of air dry
34	If fixing time not well controled, the tissue may be not good for cryosection	Depending on fixing time, the tissue may have slightly varied texure, which may influence the following cryosection and staining	In our laboratory, we recommend to incubate E9.5–E11.5 embryos for 15 min, E12.5–E18.5 hearts for 20 min, neonatal hearts for 30 min, adult hearts and other mouse organs, such as lungs, for 1 h, small intestine for 2 h
35	Tissue sections with good morphology are hard to produce	The pre-colding time may influence the quality of cryosection	We recommend incubate adult liver tissues at 4 °C for 1 h, it may takes longer incubating time for other tissues like lung
38	Antibodies do not work	Tissue sections have been stored for protracted periods of time; The concentration of antibodies is not optimized	Do not keep the section for too long for immunostaining; Refer to the concentration of antibodies
40	The dye on either nucleus or cytoplasm is exceedingly deep	The incubating time in hematoxylin A and Eosinn-Y solution is not coordinated well	It is recommended to prestain a single section for a refenrence. According to the dying effect of prestaing, adjust the incubating time to reach a balance. The dying effect may vary depending on the batch or used expiry of the reagents

27 Generate a melt curve using the Applied Biosystems real-time PCR system software. Compare the CT of an unknown sample against a standard curve with known copy numbers to obtain absolute quantitation.

### Western blot [TIMING ~2 days]

28 Western blot of FAH across *Fah-LSL/+*, *Fah-LSL/LSL*, and *Fah-LSL/LSL*;*ACTB-Cre* mice.

(A) Collect liver tissues at predetermined stages.

(B) Homogenize liver tissue samples in cold RIPA lysis buffer (Beyotime, P0013B) that contains protease inhibitors (Roche, 11836153001).

(C) Incubate on ice for 30 min to facilitate complete lysis and then centrifuge at 13,500 *g* at 4 °C for 5 min. Carefully transfer the supernatant to a new tube, avoiding the pellet.

(D) Use a protein assay kit to quantify the protein concentration in the supernatants. Normalize the protein concentrations and mix with loading buffer (Beyotime, p0015L).

(E) Boil samples for 10 min to denature proteins.

(F) Prepare the gel electrophoresis apparatus by placing precast gradient gels in the system (Beyotime, P0469M). Load an equal amount of protein into each well of two separate gels for FAH and GAPDH. Run the gel at 100 V until the dye front reaches the end.

(G) Pre-wet the polyvinylidene fluoride membranes (Millipore, IPVH00010) in methanol for 5 min. Assemble the gel and membrane in a transfer sandwich. Perform protein transfer at 100 V for 1 h or until completion.

(H) Wash membranes in TBST for 5 min. Block the membranes in TBST containing 5% BSA on an orbital shaker at room temperature for 1 h.

(I) Dilute the primary antibodies of FAH (Abclonal, A13492; 1:500) and GAPDH (Proteintech, 60004-1-IG; 1:2000) in TBST containing 1% BSA.

(J) Incubate the membranes at 4 °C overnight with gentle agitation.

(K) Wash thrice in TBST for 10 min.

(L) Incubate with HRP-conjugated secondary antibodies (Thermo Fisher Scientific, WBKLS0500) at room temperature for 1 h.

(M) Wash the membranes in TBST thrice for 10 min.

(N) Apply chemiluminescent HRP substrate to the membranes.

(O) Capture images using a chemiluminescence imaging system.

**[TIP]** Always wear appropriate personal protective equipment. Optimize antibody concentrations and incubation times based on preliminary experiments.

29 Maintain *Fah-LSL/LSL* mice on NTBC-supplemented water until they were eight weeks old. Terminate NTBC administration and measure the body weight of *Fah-LSL/LSL* mice and *Fah-LSL/+* littermates two weeks after NTBC withdrawal. Record the observations of any moribund state in *Fah-LSL/LSL* mice by the 8th week post-NTBC withdrawal.

30 Conduct a histological analysis of the livers of *Fah-LSL/+*, *Fah-LSL/LSL*, and *Fah-LSL/LSL*;*ACTB-Cre* adult mice to assess hepatic architecture and identify any extensive liver damage throughout the liver lobules (see Steps 32–37 for immunofluorescence staining and Step 38 for H&E staining).

### Characterization of the *CK19-CreER;R26-tdT;Fah-LSL/LSL* mouse line

31 Cross the *CK19-CreER*;*R26-tdT;Fah-LSL/+* mouse line with the *Fah-LSL/LSL* line to trace BECs during liver injury. Breed the mice as described in Step 16. Conduct a genotyping test as described in Steps 4–15.

32 In this assay, supply NTBC-containing water (7.5 μg/mL) until eight weeks after birth. Then, 14 days before NTBC withdrawal, administer 0.2 mg of TAM per gram mouse body weight by oral gavage four times; administer an equal amount of oil to the control group. On day 28 after NTBC withdrawal, reintroduce NTBC for 5 days.

**[TIP]** NTBC reintroduction is necessary to ensure survival and enable this regenerative process to compensate for the fulminant liver failure in our model compared with mice without NTBC introduction.

33 Conduct serum biochemical analysis for liver function.

(A) Collect blood from the indicated mice and centrifuge at 850 *g* at 4 °C for 15 min.

(B) Collect the supernatant and mix it gently with the thawed ALT or AST reagents.

(C) Load the reagents and control solutions into the designated slots in VITROS 4600.

(D) Compare the ALT and AST levels under different conditions (TAM or oil administration).

34 Collect samples at the designed experimental time for cryosection and staining [TIMING ~3 h].

(A) Fix the tissue samples with 4% (*w*/*v*) paraformaldehyde (PFA) at 4 °C for 15 min to 2 h, depending on tissue size and type.

**[?TROUBLESHOOTING]**

(B) Wash the tissue samples with cold PBS thrice (15 min each time).

(C) Prepare a Petri dish with 1% (*w*/*v*) agarose dissolved in distilled water. Place the samples on the dish. Dig a small hole in the agarose gel to stabilize the irregularly shaped tissue.

(D) Place the tissue sample in the hole to keep it stable and in the desired orientation. Take the bright-field and fluorescent views of the images as detailed in the operating manual for your specific stereoscopic microscope.

**[TIP]** When dealing with mice that might express different fluorescent proteins, use distinct excitation wavelengths. In our experiments, only tdT might be expressed; as such, take bright-field and RFP channels to capture the whole-mount image.

35 Embed samples for cryosection and staining [TIMING ~14 h].

(A) Place the samples (after fixation and whole-mount imaging) in 30% (*w*/*v* in PBS) sucrose at 4 °C overnight to dehydrate the samples.

**[TIP]** Dehydration time is dependent on tissue size. In our laboratory, the adult tissues were dehydrated overnight. If the samples were not fully dehydrated, the freezing deformation would affect the section morphology.

(B) After liquid aspiration, place the sample in a cryogenic mold and then add the OCT (Sakura) compound to cover the tissue entirely.

(C) Leave the tissue samples in the OCT compound at 4 °C for 30 min–1 h.

**[?TROUBLESHOOTING]**

(D) Adjust the orientation of the samples. Try to keep the tissue in the center of the cryogenic mold without touching the edge to maintain sample integrity when sectioned.

(E) Freeze the tissues on the freezing metal stage in the cryostat (temperature set to −20 °C). Do not leave the block (sample frozen in the mold) in the cryostat overnight to avoid drying out.

(F) The frozen tissue blocks can be safely stored at −80 °C for several months.

36 Tissue cryosection. Set the cryostat temperature to −20°C. Insert and fix the cryostat blade and glass. Add some OCT compound to the specimen chuck first; then, remove the tissue samples from the cryogenic mold and place them on the specimen chuck. Transfer the specimen chuck with the tissue samples to the freezing stage of the cryostat; the samples will stick to the specimen chuck after OCT compound freezing. Section the tissue samples at 10–20 μm thickness. Collect the samples on the positively charged slides.

**[TIP]** Before placing both of your hands in the cryostat, remember to lock the rocker bar to prevent injury by the cryostat blade.

**[TIP]** To determine the labeling efficiency of *CK19-CreER*, researchers should attempt to perform serial sectioning; that is, the whole tissue should be sectioned, and all sections should be collected. Unless this approach is implemented, the data collected as described in the rest of the Procedure section may not accurately reflect the labeling efficiency as a proportion of the labels would be left undetected in discarded tissue samples.

37 The slides can be used immediately or stored at −20 °C for several weeks to several months.

38 Immunofluorescence staining [TIMING ~14 h].

(A) Place the cryosections in 50 mL of PBS at room temperature for 5 min to remove the OCT compound from the tissue samples.

(B) Add ~300 µL of blocking PBSST solution to each slide and leave it to act at room temperature for 30 min.

(C) After removing the blocking PBSST solution from each slide, add primary antibody diluted in ~300 µL working PBSST solutions to each slide at the appropriate dilution ratio, and incubate the slides at 4 °C overnight.

**[TIP]** If examination of two or more different antigens in the same sample is required at the same time, ensure that primary antibodies (and their corresponding secondary antibodies) are available from different species.

(D) Wash the slides thrice (5 min each time) with PBS.

(E) Add ~300 µL of secondary antibody solution in working PBSST solutions to each slide and incubate the slides at room temperature for 30 min in the dark.

**[TIP]** Protect the solution from light to prevent quenching of the secondary antibody fluorescence. The secondary antibody should correspond to the species of the primary antibody.

(F) Wash the slides thrice (5 min each time) with PBS.

(G) Mount the slides with the mounting medium containing the nuclear dye DAPI.

**[TIP]** Although the mounted slides could be stored at −20 °C for up to one week, collect the fluorescence images as soon as possible, or the fluorescence signal would be destroyed.

**[?TROUBLESHOOTING]**

39 Use the confocal microscope to acquire the images of immunostained tissue sections. For this purpose, start by browsing all the stained slides under low magnification (*e*.*g*., ⩽4×) by using a stereoscopic microscope. Subsequently, choose a random field from which to record images under a confocal microscope. Record low- (10×) and high- (40× or 60×) magnification confocal images.

40 H&E staining and imaging.

**[TIP]** All the steps in H&E staining should be done in the fuming cupboard.

(A) Place the cryosections in 50 mL of PBS at room temperature for 5 min to remove the OCT compound from the tissue samples.

(B) Incubate the cryosections in hematoxylin A for 10 min.

(C) Rinse and agitate the cryosections in tap water 2–3 times.

(D) Incubated in 1% concentrated hydrochloric acid diluted in 70% ethanol for 1 min.

(E) Rinse and agitate the cryosections in tap water 2–3 times.

(F) Incubate in bluing reagent (1% ammonia) for 1 min.

(G) Rinse and agitate the cryosections in tap water 2–3 times.

(H) Rinse the cryosections in 95% ethanol for 10 s.

(I) Stain the sections with Eosin-Y solution for 10 s.

(J) Rinse the cryosections in 95% ethanol for 10 s.

(K) Rinse the cryosections in 100% ethanol for 4 min.

(L) Rinse the cryosections in xylene for 10 min.

(M) Mount the sections with a resinous medium.

(N) Capture the image with an Olympus microscope.

**[?TROUBLESHOOTING]**

### Utilizing the dual genetic lineage tracing approach to study the behavior of CK19+ HNF4α+ TLPCs

41 Cross the *CK19-CreER*;*Fah-LSL/LSL;R26-RL-tdT* mouse line with the HNF4a-DreER;*Fah-LSL/LSL* mouse line to further study the characterization of TLPCs. Mouse breeding is performed as described in Step 16. Genotyping test was conducted as described in Steps 4–15.

42 In this assay, a low dosage of TAM (0.05 mg TAM per gram mouse body weight) was administered on day 20 after NTBC removal. *CK19-CreER*;*Fah-LSL/LSL;R26-RL-tdT* or *HNF4α-DreER;Fah-LSL/LSL;R26-RL-tdT* mice were used as controls. By collecting samples on day 25 after NTBC removal, we confirmed that R26-RL-tdT could only be activated by dual recombination. (See Steps 34–39 for immunofluorescence staining).

43 Clonal analysis. Through low-dosage TAM administration, we achieved sparse labelling and later clonal analysis at seven weeks post low-dose TAM. The sparsity of the labeling enables precise tracking and detailed characterization of cellular clones over time without complications that might arise in more densely labeled systems.

## ANTICIPATED RESULTS

**Figure 2:**
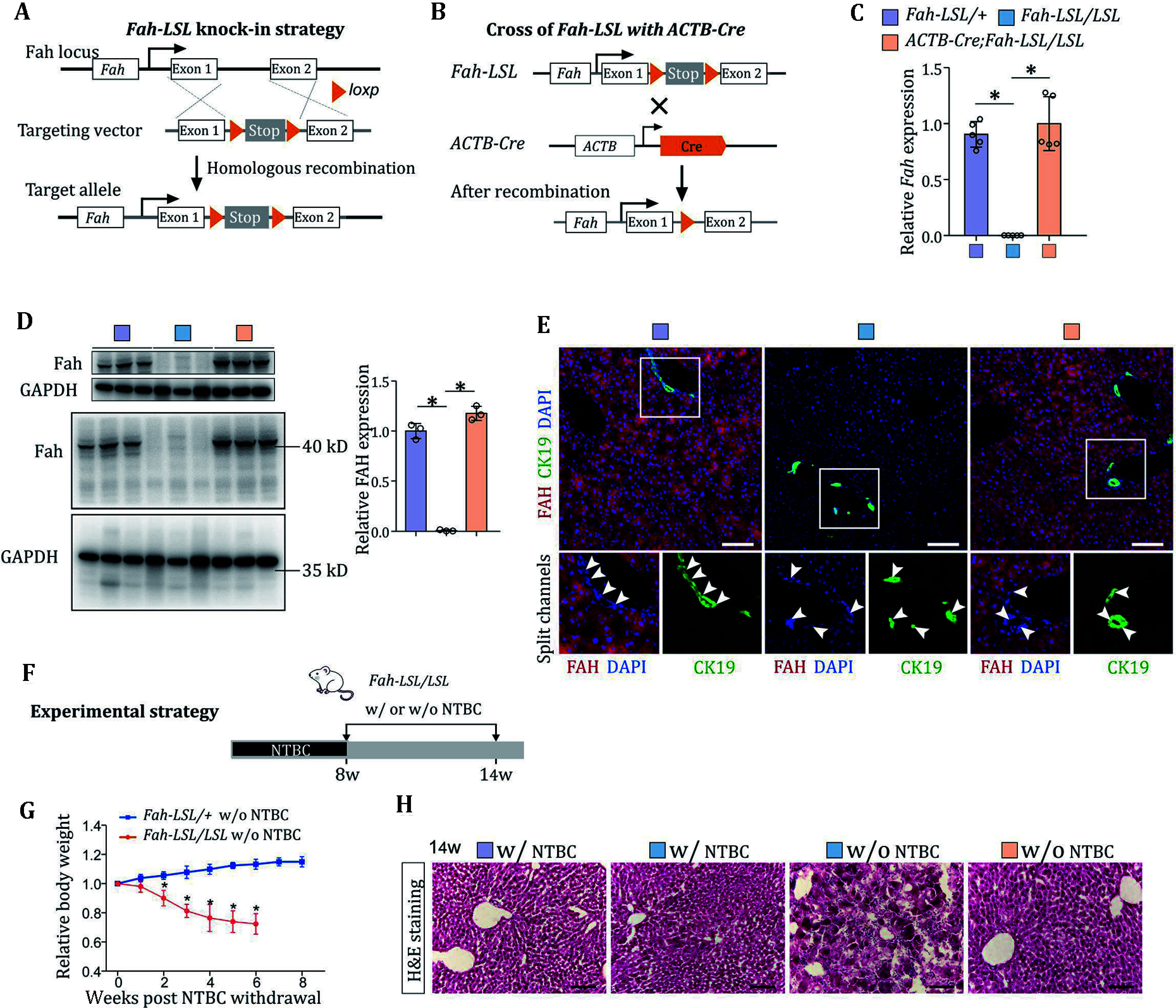
Generation and characterization of Fah-LSL mice. **A** Schematic diagram showing the strategy for generation of Fah-LSL knock-in allele by homologous recombination. **B** Schematic diagram showing re-expression of Fah after LSL removal by crossing of Fah-LSL mice with ACTB-Cre mice. **C** Relative mRNA expression levels of Fah in the livers of adult Fah-LSL/+, Fah-LSL/LSL and Fah-LSL/LSL;ACTB-Cre mice treated with NTBC. Data are the mean ± SD; *n* = 5 mice; *P < 0.0001, *P < 0.0001. **D** Western blotting for FAH in the livers of adult Fah-LSL/+, Fah-LSL/LSL and Fah-LSL/LSL;ACTB Cre mice treated with NTBC. Quantification of the relative protein levels of FAH was shown on the right. Data are the mean ± SD; *n* = 3 mice; *P < 0.0001, *P < 0.0001. Statistical analysis in Panels C and D was performed by ANOVA followed by Bonferroni test for multiple comparisons and adjustments were made for multiple comparisons. **E** Immunostaining for FAH and CK19 on the liver sections from adult Fah-LSL/+, Fah-LSL/LSL and Fah-LSL/LSL;ACTB-Cre mice treated with NTBC. Arrowheads, CK19+ FAH- BECs. Scale bar,100 µm. **F** Schematic showing the experimental strategy for NTBC withdrawal (w/o NTBC) and tissue analysis at indicated time points. **G** Weekly body weight measurements for Fah-LSL/+ and Fah-LSL/LSL mice after NTBC withdrawal (w/o NTBC). The data are normalized to the body weights at week 0. Data represent mean ± SD; *n* = 5 mice; week2: *P < 0.0004; week3–week6: *P < 0.0001. Statistical analysis was performed by multiple *t*-test. Each row was analyzed individually without assuming a consistent SD. **H** Hematoxylin and eosin (H&E) staining of liver sections. Scale bar, 100 µm

Following this protocol, we generated mice carrying the *Fah-LSL* allele (Fig. 2A). By crossing *Fah-LSL/+* mice with *Fah-LSL/+* and *ACTB-Cre* mice, we constructed *Fah-LSL* homozygous mice and *Fah-LSL/LSL*;*ACTB-Cre* mice (Fig. 1B). To verify whether *Fah* was not expressed in *Fah-LSL/LSL* mice and whether *Fah-LSL/LSL*;*ACTB-Cre* mice restored the *Fah* expression, we examined the relative mRNA expression levels and protein levels of *Fah* in livers by quantitative RT-PCR and western blot, respectively (Figs. 2C and 2D). We also validated this finding through immunofluorescence staining (Fig. 2E). Subsequently, we examined the phenotypes of *Fah-LSL/LSL* mice after the cessation of NTBC administration. The *Fah-LSL/LSL* mice were sustained on NTBC-supplemented water until they reached eight weeks of age. In a comparative analysis with their *Fah-LSL/+* littermates, *Fah-LSL/LSL* mice manifested a notable reduction in body weight within two weeks post-NTBC withdrawal and died by the 8th week (Figs. 2F and 2G). The histological assessment of the livers from *Fah-LSL/LSL* mice revealed aberrant hepatic architecture and extensive hepatic damage throughout the liver lobules (Fig. 2H). These findings demonstrated that our *Fah-LSL* mouse model accurately recapitulated the prototypical pathological phenotypes observed in *Fah*-null mice, characterized by acute hepatic failure and compromised hepatocellular regenerative capacity. Additionally, our *Fah-LSL* knockout allele provided the methodological advantage of allowing the Cre-mediated restoration of *Fah* expression.

**Figure 3:**
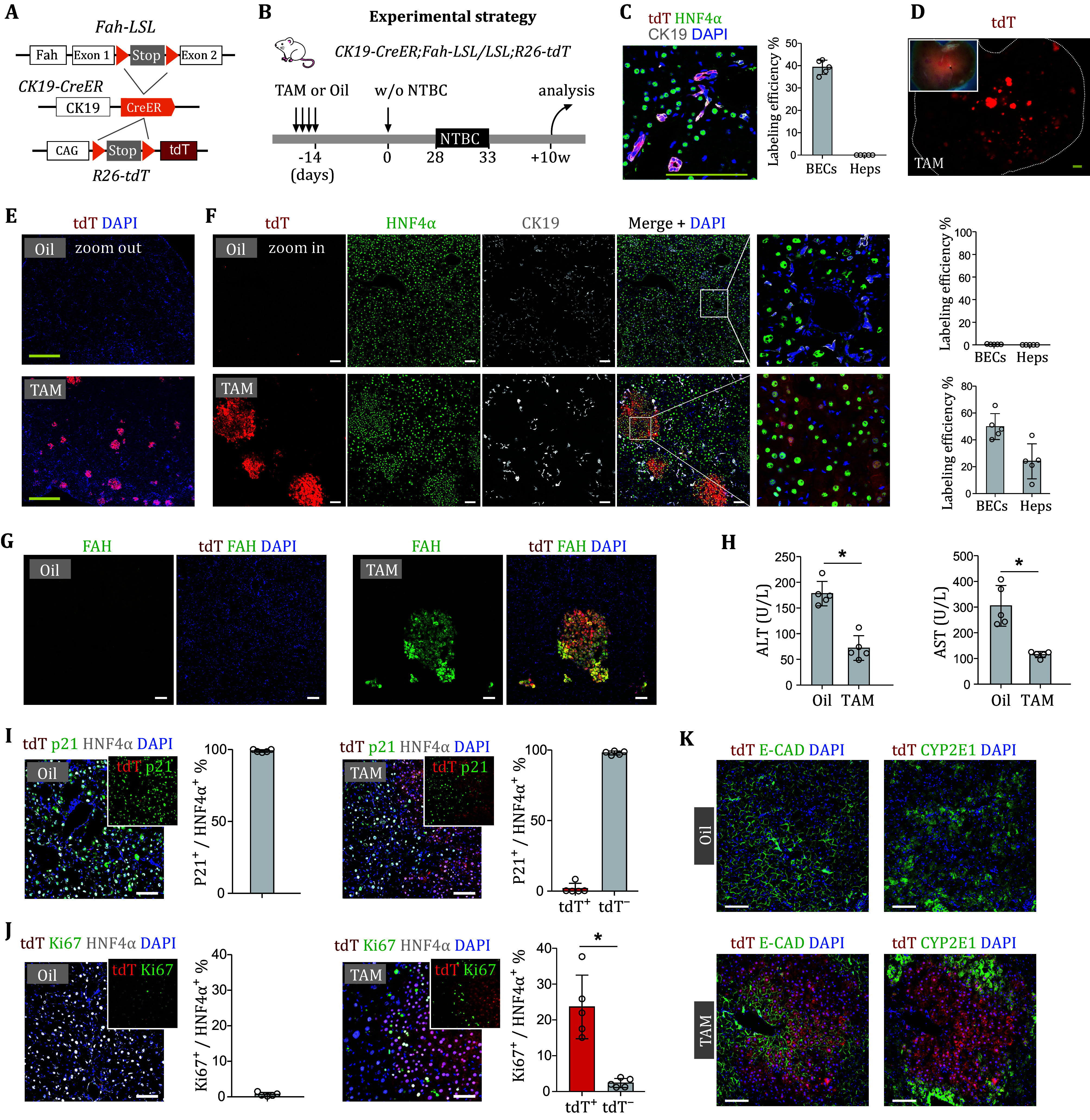
BEC-derived hepatocytes contribute to liver regeneration. **A** Schematic diagram showing the experimental design for recovery of Fah gene in BECs and lineage tracing. **B** Schematic diagram showing the experimental strategy of lineage tracing in CK19-CreER;Fah-LSL/LSL;R26-tdTmice. **C** Immunostaining for tdT, CK19, and HNF4α on liver sections collected at day 0. Scale bar, 1 mm. Right panel shows quantification of cell labeling efficiency. Data represent mean ± SD; *n* = 5 mice. **D** Whole-mount fluorescence liver images. Scale bars, 1 mm. **E** Immunostaining for tdT on liver sections from mice treated with TAM or oil. Scale bars, 1 mm. **F** Immunostaining for tdT, HNF4α, and CK19 on the liver sections from mice treated with TAM or oil. Quantification of percentage of tdT+ BECs and tdT+ Heps is shown in the adjacent graph. Data represent mean ± SD; *n* = 5 mice. Scale bars, 100 µm. **G** Immunostaining for tdT and FAH on the liver sections from mice treated with TAM or oil. Scale bars, 100 µm. **H** Serum ALT and AST of mice treated with TAM or oil. Data are the mean ± SD; *n* = 5 mice. *P < 0.0001; *P < 0.0001. **I**, **J** Immunostaining for tdT, HNF4α and p21 (**I**) or Ki67 (**J**) on liver sections. Inserts show green and red fluorescence channels. Quantification of Ki67 or p21 staining in tdT+ and tdT– hepatocytes is shown in the adjacent graph. Data represent mean ± SD; *n* = 5 mice. *P < 0.0001; Statistical analysis in Panels H and J was performed by two-tailed unpaired Student’s *t*-test. **K** Immunostaining for tdT with E-CAD or CYP2E1 on the liver sections from mice treated with TAM or Oil. Scale bars, 100 µm

To trace BECs in our liver injury model, we generated *CK19-CreER*;*Fah-LSL/LSL*;*R26-tdT* mice (Fig. 2A), whose tdTomato (tdT) was induced and *Fah* was restored in BECs after tamoxifen (TAM)-induced Cre-loxP recombination. To test the specificity of tracing BECs in our system, we collected liver sections on day 0 and found that only BECs (~40%) but not hepatocytes were specifically labeled before injury (Figs. 3B and 3C). When collecting the samples ten weeks after NTBC withdrawal, we observed many round-shaped tdT+ clones in TAM-treated *CK19-CreER*;*Fah-LSL/LSL*;*R26-tdT* livers (Fig. 3D), and tdT was detected in hepatocytes (~25%; Figs. 3E and 3F). Notably, all tdT+ hepatocytes were positive for FAH in TAM-treated mouse livers (Fig. 3G), and the liver injury was mitigated (Fig. 3H). These tdT+ hepatocytes did not express p21 and showed an increased proliferation compared with tdT– hepatocytes (Figs. 3I and 3J). Large clones of tdT+ hepatocytes re-established metabolic zonation by expressing periportal and pericentral zonation markers in the respective lobular zones (Fig. 3K). These results showed that the *CK19-CreER*;*Fah-LSL/LSL*;*R26-tdT* mouse line was successfully constructed to study BEC-to-hepatocyte transdifferentiation.

**Figure 4:**
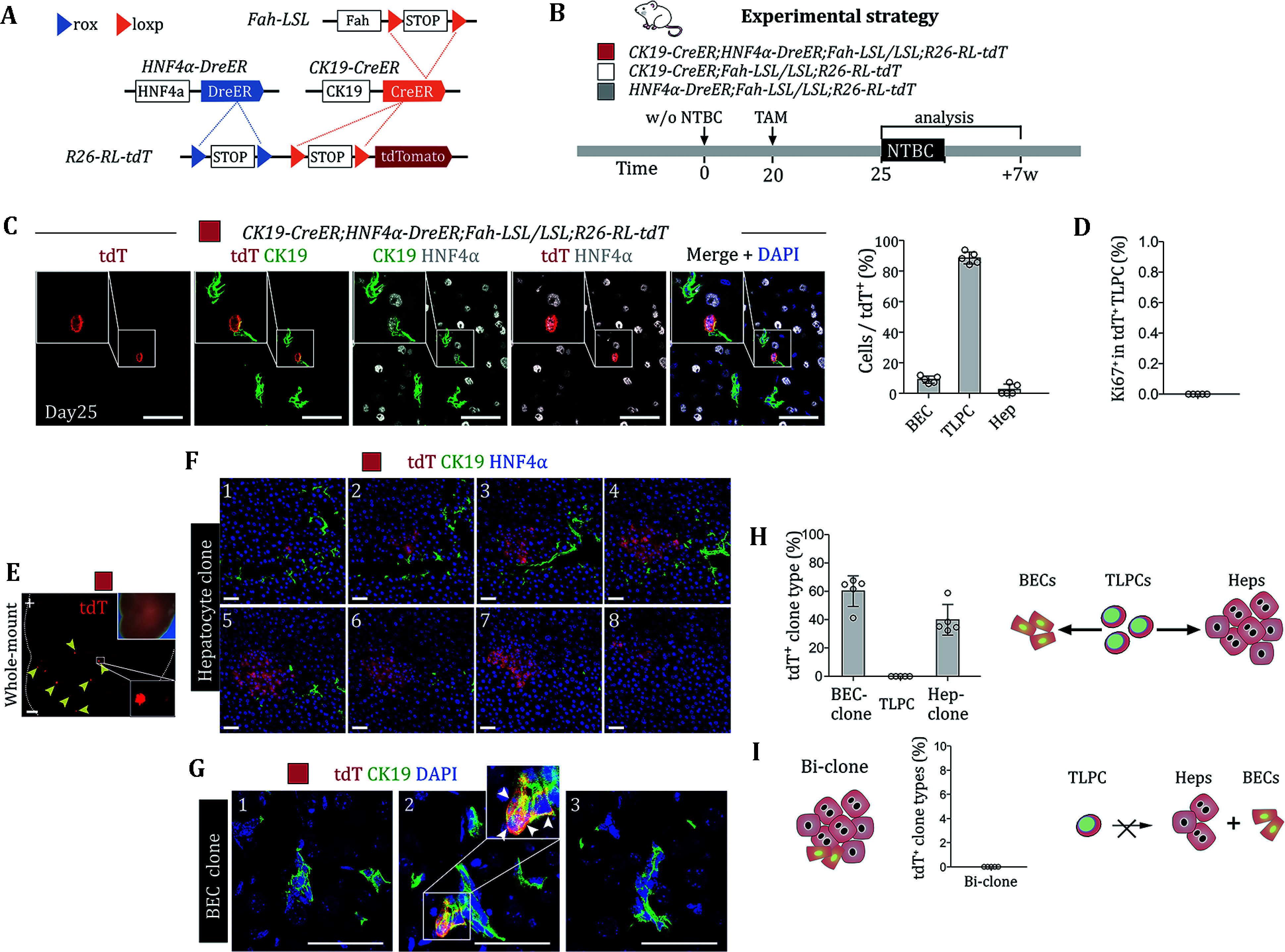
Biopotent TLPCs generate hepatocytes or adopt BEC fate during liver repair. **A** Schematic showing the strategy for TLPC lineage tracing. **B** Schematic showing experimental strategy. **C** Immunostaining for tdT, HNF4α and CK19 on liver sections collected at day 25 from CK19-CreER;HNF4α-DreER;Fah-LSL/LSL;R26-RL-tdT mice. Scale bars, 50 µm. Right panel shows the percentage of BECs, TLPCs and hepatocytes in tdT+ cells. Data represent mean ± SD; *n* = 5 mice; total 289 tdT+ cells were counted (BECs: 25; TLPCs: 258; hepatocyte: 6). **D** Percentage of Ki67+ cells in tdT+ TLPCs. Data represent mean ± SD; *n* = 5 mice. **E** Whole-mount tdT fluorescent liver images from indicated mice at 7 weeks after the first NTBC removal. Bright field images are shown as inserts. Arrows indicate tdT+ clones. Scale bars, 1 mm. **F** Immunostaining for tdT, HNF4α and CK19 on serial sections (20 µm) of livers collected at week 7 from CK19-CreER;HNF4α-DreER;Fah-LSL/LSL;R26-RL-tdT mice. Scale bars, 50 µm. **G** Immunostaining of tdT, and CK19 on serial liver sections (20 µm) collected at week 7 from CK19-CreER;HNF4α-DreER;Fah-LSL/LSL;R26-RL-tdT mice. Scale bars, 50 µm. White arrows indicate tdT+ BECs. **H** Percentage of tdT+ clones containing BECs, TLPCs and hepatocytes. Schematic on the right panel showing that TLPCs could give rise to hepatocytes and revert back to BECs. Data represent mean ± SD; *n* = 5 mice; total 480 tdT+ clones were counted. **I** Percentage of hybrid clone that consists of BECs and hepatocytes. Schematic showing that TLPCs could not give rise to hepatocytes and BEC simultaneously. Data represent mean ± SD; *n* = 5 mice

To further characterize BEC-derived TLPCs, we developed a Cre-loxP and Dre-rox-based dual genetic lineage tracing approach to indelibly label CK19+ HNF4α+ TLPCs. We generated *CK19-CreER*;*HNF4α-DreER;Fah-LSL*/*LSL;R26-RL-tdT* mice, whose tdT could be expressed only after the TAM-induced removal of two Stop sequences by both Dre-rox (HNF4α+) and Cre-loxP (CK19+) recombinations (Fig. 4A). We treated the mice with a low dosage of TAM on day 20 after NTBC removal and collected livers on day 25 and week 7 (Fig. 4B). We found that 88.61 ± 3.80% of tdT+ cells were TLPCs (CK19+ HNF4α+), 8.91 ± 2.19% of tdT+ cells were BECs (CK19+ HNF4α–), and 2.48 ± 3.48% of tdT+ cells were hepatocytes (CK19–/HNF4α+) in *CK19-CreER*;*HNF4α-DreER;Fah-LSL/LSL;R26-RL-tdT* mouse livers (Fig. 4C). Notably, none of tdT+ TLPCs were Ki67+ (Fig. 4D), indicating TLPC quiescence. We next assessed the lineage conversion potential of individual TLPCs over time at seven weeks post low-dose TAM, enabling clonal analysis. We observed sporadic tdT+ clones consisted of either hepatocytes (CK19– HNF4α+) or BECs (CK19+ HNF4α–); however, we did not find tdT+ TLPCs (CK19+ HNF4α+; Figs. 4E–4G). Furthermore, 60.14 ± 10.80% of the tdT+ clones were BEC clones, and 39.86 ± 10.80% of the tdT+ clones were hepatocyte clones (Fig. 4H), suggesting that TLPCs contribute to BEC or hepatocyte lineages during liver regeneration. While TLPCs are bipotent, none of the individual tdT+ clones consisted of both hepatocytes and BECs (Fig. 4I). Collectively, these data indicated that bipotent TLPCs could either transdifferentiate into hepatocytes or redifferentiate into BECs during liver regeneration.

## MATERIALS

### Biological materials

Mice. Experiments involving mice were performed in strict compliance with the protocols sanctioned by the IACUC at the Center for Excellence in Molecular Cell Science, a division of the Shanghai Institutes of Biological Sciences under the Chinese Academy of Science. The ethical approval for these animal studies was granted under the protocol number SIBCB-S374-1702-001-C1.

### Reagents

• NTBC (Shanghai Xuewu Biotech, cat. no. CTL-800-661)

• Isopropanol (SinoPharm, cat. no. 64-63-0)

• Ethanol (SinoPharm, cat. no. 64-17-5)

• Distilled water (Invitrogen, cat. no. 10977023)

• Tris (Amresco, cat. no. 77-86-1)

• HCl (SinoPharm, cat. no. 7467-01-0)

• SDS (SinoPharm, cat. no. 751-21-3)

• NaCl (SinoPharm, cat. no. 7647-14-5)

• Tween 20 (Thermo Fisher Scientific, cat. no. 003005)

• Proteinase K (Roche, cat. no. 3115852001)

• 2× Taq PCR mix (Vazyme, cat. no. p213-03)

• Gel-Red (Tanon, cat. no. 170-3001BE)

• Agarose (Tsingke Biotechnology, cat. no. TSJ001)

• PBS (Invitrogen, cat. no. C10010500BT)

• Nucleic acid dye (Promega, cat. no. PRH1181)

• OCT compound (Sakura, cat. no. 1S-LB-4583-EA)

• Triton X-100 (SinoPharm, cat. no. 9002-93-1)

• Paraformaldehyde (PFA; Sigma, cat. no. P6148-1KG)

• Sucrose (Invitrogen, cat. no. 15503022)

• Normal donkey serum (Jackson Immunoresearch, cat. no. 017-000-121)

• Tamoxifen free base (Sigma, cat. no. T5648-5G)

• Corn oil (supermarket, edible oil)

• RIPA lysis buffer (Beyotime, cat. no. P0013B)

• Protease inhibitors (Roche, cat. no. 11836153001)

• Loading buffer (Beyotime, cat. no. p0015L)

• Precast gradient gels (Beyotime, cat. no. P0469M)

• Polyvinylidene fluoride (PVDF) membranes (Millipore, cat. no. IPVH00010)

• Bovine Serum Albumin (SSCB, cat. no. H2N0611)

• Chemiluminescent HRP substrate (Thermo Fisher Scientific, cat. no. WBKLS0500)

• TRIzol Reagent (Invitrogen, cat. no. 15596018)

• Prime Script RT kit (Takara, cat. no. RR047A)

• Power SYBR Green PCR Master Mix (2X) (Thermo Fisher Scientific, cat. no. 4367659)

• Western antibodies

• FAH (Abclonal, A13492; 1:500)

• GAPDH (Proteintech, 60004-1-IG; 1:2,000)

• Peroxidase AffiniPure Goat Anti-Rabbit IgG (Jackson ImmunoResearch, 111-035-047; 1:4,000)

• Peroxidase AffiniPure Donkey Anti-Mouse IgG (Jackson ImmunoResearch, 715- 035-150; 1:4,000).

• Weigert's iron hematoxylin kit (Sigma-Aldrich, cat. no. 115973)

• Immunostaining antibodies

• DAPI (Invitrogen, cat. no. D21490)

• tdT (Rockland, cat. no. 600-401-379, 1:500)

• p21 (Abcam, cat. no. ab188224; 1:500)

• Ki67 (Abcam, cat. no. ab15580; 1:200)

• CK19 (Developmental Studies Hybridoma Bank, cat. no. TROMA-III, 1:500)

• HNF4α (Cell Signalling, cat. no. 3113s; 1:500)

• FAH (Abclonal, cat. no. A13492; 1:500)

• GS (Abcam, cat. no. Ab49873; 1:1,000)

• E-cadherin (E-cad, cat. no. Cell signaling, 3195; 1:100)

• CYP2E1 (Abcam, cat. no. ab28146; 1:100)

• HRP-conjugated antibodies with ImmPACT DAB kit (Vector lab, cat. no. SK-4105)

• Donkey anti-rabbit antibody Alexa Fluor 488 (Thermo Fisher Scientific, cat. no. A-21206; RRID: AB_2535792)

• Donkey anti-rabbit antibody Alexa Fluor 555 (Thermo Fisher Scientific, cat. no. A-31572; RRID: AB_162543)

• Donkey anti-goat antibody Alexa Fluor 647 (Thermo Fisher Scientific, cat. no. A-21447; RRID: AB_2535864)

• Donkey anti-rat antibody Alexa Fluor 647 (Abcam, cat. no. ab150155; RRID: AB_2813835)

### Reagent setups

• 1 mol/L Tris-HCl (pH 7.8). In this procedure, 60.57 g of Tris base was dissolved into 450 mL of distilled water and pH was adjusted to 7.8 by adding concentrated HCl to prepare ~500 mL of 1 mol/L Tris-HCl. Then, a 500 mL solution was made using distilled water. The solution could be stored at room temperature (20 ± 5 °C).

• Lysis buffer. The reagents listed in Table 4 were combined to prepare the lysis buffer for DNA extraction. The lysis buffer was prepared without proteinase K and stored at room temperature for about six months. Proteinase K was stored at −20 °C and directly added to the lysis buffer before use.

**Table 4:** The reagents list

Components	Final concentration
Tris-HCL (PH 7.8)	100 mmol/L
EDTA	5 mmol/L
SDS	0.2% (*v*/*v*)
NaCl	200 mmol/L
Proteinase K	100 μg/mL

• TAE buffer. In this procedure, 1 L of 50× TAE buffer was prepared for running DNA gels. First, 242 g of Tris base and 18.612 g of EDTA were dissolved in distilled water. Then, 57.1 mL of glacial acetic acid was added to the homogenized solution. pH was adjusted to 8.3 by using NaOH, and distilled water was added to obtain 1 L of the buffer. This buffer could be stored at room temperature for about six months. Then, 50× TAE buffer was diluted to 1× with distilled water just before use.

• 1% (*w*/*v*) agarose gel. In this procedure, 1 g of agarose powder was added to 100 mL of 1× TAE buffer and then heated in a microwave oven to dissolve until the solution was homogeneous. Then, 10 μL of 10,000× Gel-Red was diluted in the solution, mixed thoroughly, and cast.

• 10x TBS stock. In this procedure, 242.28 g of Tris base and 584.4 g of NaCl were weighed. Approximately 800 mL of distilled water was added to a 1 L glass beaker. The weighed Tris base and NaCl were added to water while stirring with a magnetic stirrer. pH was adjusted to 7.4–7.6 by adding concentrated HCl or NaOH, if necessary. After the pH was stable, the total volume was reduced to 1 L with distilled water. Then, the 10× TBS stock solution was stored at room temperature for short-term use or at 4 °C for long-term storage.

• 1× TBST. In this procedure, 100 mL of 10× TBS stock solution was taken and diluted to 1 L with distilled water to make 1× TBS. Then, 0.5 mL of Tween 20 was added to 1 L of 1× TBS to achieve a final concentration of 0.05% (*v*/*v*).

• PBST solution. In this procedure, 49.9 mL of PBS (pH 7.4) was mixed with 100 µL of Triton X-100. This solution could be safely stored at 4 °C for several weeks.

• Blocking PBSST solution. Normal donkey serum was dissolved in PBST at a concentration of 5% (*v*/*v*). This solution was freshly prepared before use.

• Staining PBSST solution. Normal donkey serum was dissolved in PBST at a concentration of 2.5% (*v*/*v*). This solution was freshly prepared before use.

• Tamoxifen solution (20 mg/mL). In this procedure, 0.2 g of tamoxifen powder was dissolved into 10 mL of corn oil in a 15 mL Corning centrifuge tube by shaking overnight at room temperature. The tube was wrapped with foil because tamoxifen is light sensitive. The solution was sterilized by filtering with a 0.22 μm filter and stored at 4 °C for ⩽1 month.

### Equipment

• PCR thermal cycler (Bio-Rad, cat. no. S1000)

• Ultraviolet detector (Wealtec, Dolphin-Doc Plus)

• Dolphin 1D software (Wealtec)

• Cryostat (Thermo Scientific, cat. no. HM525)

• Confocal microscope (Olympus, model no. FV1200, or Zeiss, model no. LSM710)

• Stereoscopic microscope (Zeiss, model no. AxioZoom V16, Olympus, model no. BX53)

• QuantStudio 6 Real-Time PCR System (Thermo Fisher Scientific)

• 4600 fully automatic biochemical analyzer (VITROS)

• Water bath (Fisher Scientific, model no. ISOTEMP 210)

• Centrifuge (Eppendorf, cat. nos. 5810R and 5424R)

• Gavage needle (FST, cat. no. 18061-24)

• Microscope cover glass (Fisher Scientific, cat. no. 12-548-5M)

• Microscope glass slides (Fisher Scientific, cat. no. 12-550-17)

• Cryogenic mold (Sakura, cat. nos. 4566 and 4565)

• Specimen chuck (Thermo Scientific, cat. no. 715220-30)

• Cryostat blade (Feather, cat. no. 21030294)

• ImmEdge Pen (Vector, cat. no. H4000)

• 50-mL Corning centrifuge tube (Sigma, cat. no. CLS430829)

• 0.22-μm filter (Merck Millipore, cat. no. GVWP04700)

• 15-mL Corning centrifuge tube (Sigma, cat. no. 430791)

• 0.2-mL PCR tube (Axygen, cat. no. 321-02-051)

• Heat mat (Globalebio, cat. no. GE0-20W)

• 1-mL syringe (Shanghai Kindly Group)

## Conflict of interest

Xingrui Wang, Wenjuan Pu, Huan Zhu, Mingjun Zhang and Bin Zhou declare that they have no conflict of interest.
